# Remarks on Southern Cattle Fever1Read before the Virginia State Veterinary Medical Association, Charlottesville, Va., January 3, 1895.

**Published:** 1895-02

**Authors:** W. H. Harbaugh


### REMARKS ON SOUTHERN CATTLE FEVER.[1]

BY W. H. HARBAUGH, V.S.

TEXAS fever, southern cattle fever, or, as it is better known to the public in this State, murrain or bloody murrain, is a subject of vast importance to every member of this Association, as we all reside and practise either within the territory designated by the United States authorities as the permanently infected district, or close to the so-called line of the infected district, where cases of the disease are liable to occur at any time during the season of the year the fever manifests itself, and we are, therefore, called upon to treat the disease as practical veterinarians, not as investigators or theorists.

The cattle-owner does not care how well we are read up on the subject; the difference in opinion as to the real cause of the malady is nothing to him; his animal is suffering with a disease generally supposed to be fatal, and he wants it relieved. We are called upon to relieve it, and we must do our best under the circumstances, or else acknowledge that we do not know enough about this particular disease to attempt treatment. That the disease is the least understood and the most generally misunderstood by the profession at large, and the public as well,

[1] Read before the Virginia State Veterinary Medical Association, Charlottesville, Va., January 3, 1895.

there can be no doubt. But it by no means follows that the treatment is hopeless, as I will endeavor to demonstrate later on.

Many of the erroneous ideas in regard to the malady are the direct result of veterinary writers who, knowing nothing of the subject themselves, depend on the writings of others for their knowledge, and usually fail to understand what they read. As an example of such ideas, I will quote from an article in the December *Review:*

"The micro-parasite is always present in southern cattle, even if they have been away from the southern pastures for a few years. All southern calves go through a natural inoculation by the ticks, at which age the death-rate is very small; when I say all southern calves, I think I am right, as the ticks are pretty well scattered all over the territory, when the winter is not severe enough to destroy them."

The points which I particularly desire to call your attention to at this meeting are the following:

1. The whole territory known as the permanently infected district is not infected. There are farms and sections of counties within this so-called infected area which are as free from infection as any place north of the Potomac.

2. All southern cattle are not dangerous. There are cattle raised on certain farms in the South as free from the disease as if they had been raised in any northern State, and when these cattle are taken to other farms, perhaps but a mile or two distant from where they were born and raised, they contract the disease.

3. All southern ticks are not dangerous. Right here, in the so-called permanently infected district, there are farms where ticks are on cattle at all times, and when cattle are put on the farms from outside the infected district no harm has resulted.

4. All acute cases of the disease are not hopeless ones to treat. Mild ones are favorable, and frequently recover without any treatment, and often in spite of the most atrocious treatment.

Before proceeding further, I wish it to be well understood that I am not here to criticise the work of the Bureau of Animal Industry. On the contrary, I am of the opinion that the work of the bureau stands unequalled by any similar body the world over, and should receive the hearty support and encouragement of every veterinarian who has at heart the interest of this country and his profession. But that there is room for honest difference in opinion on this subject is unquestionable; and, in fact, if you will carefully read the special report on Texas fever you will be informed by the investigators of many doubts which they freely point out.

I will also say here that so far as the disease is concerned, in my opinion, the only works of practical importance to us as veterinarians are the reports of the United States Agricultural Department and the Bureau of Animal Industry, beginning with Special Report, No. 22, 1880.

I had my first serious outbreak to contend with in the fall of 1886, on the farm of Col. A., in Chesterfield County. Many of this herd were born and raised on the farm, others were brought from Maryland and other States, added from time to time as the owner secured valuable animals to improve his herd.

About twenty of the cattle were affected with the disease, either in the mild or acute types. About six died, including a noted cow which cost her owner $3250. From the first I did not hesitate in my diagnosis of Texas fever. Post-mortem examinations confirmed my opinion. My standard authority was Dr. Salmon's article on the subject, in Special Report, No. 22. The history, symptoms, and post-mortem lesions, as given in that article, are to-day the best extant for the general practitioner, so far as I know.

Col. A. would not believe that the cattle were affected with Texas fever because they had no ticks on them. Thorough search was made for ticks, but none were found. The cattle stable was a clean, well-kept place, thoroughly ventilated and drained. The cows were cleaned daily; and when I tell you that they were cleaned with a currycomb and brush, and with water and soap, if necessary, you will understand what I mean by saying they were cleaned daily. You will also probably understand why no ticks could be discovered on them.

This was my first experience with the tick theory, which I did not believe, and as a consequence I was both amused and annoyed by the Colonel's obstinacy in regard to it, because, at that time, all I knew about the disease I learned from Special Report, No. 22, in which Dr. Salmon says:

"One of the most widely-spread opinions in regard to the causation of southern fever is the pathogenic influence of the ticks with which southern cattle are generally covered, and which migrate in large numbers to the bodies of other cattle with which they mix. But the acceptance of this view is simply an evidence of the desire of the human mind to explain the origin of mysterious phenomena. The same principle is exhibited in the popular views regarding the pathogenic nature of hollow-horn, hollow-tail, wolf-teeth, black-teeth, hooks, etc., none of which has the least foundation in fact or reason. The tick theory scarcely explains a single one of the many peculiar phenomena of the disease. Ticks are not kept from cattle by a fence. They are found everywhere, but are simply more numerous in the South. Their attacks are not confined to the latter half of the summer; nor would they be likely to remain on a pasture from spring to August, without doing harm, and then suddenly cause an outbreak of the disease. Again, the post-mortem examination plainly indicates the cause of the disease to be an agent taken into the circulation and causing the most important changes in the composition of the blood."

The foregoing disposal of the tick theory by Dr. Salmon in 1880 has much to commend even now, in 1895, notwithstanding the recent discoveries by the bureau investigators, in which the tick has occupied a prominent position.

My ideas in regard to the cause of the disease were also taken from Special Report, No. 22, in which Dr. Salmon says: "It may be doubted if the fever would attack animals unless they had eaten grass which had been soiled by the excretions of the southern animals, or, at least, which had been so near these excretions as to be contaminated by them.

Now, this idea of the cause was fully supported by the circumstances connected with the outbreak in Col. A.'s herd. Up to this outbreak there never had been a case on the farm known to any one connected with it. Many of the cattle were born and raised on the place, while cattle from the North were put on the farm at frequent intervals with no bad results. A few weeks before the outbreak some strange cattle broke through the fence and had pastured half-way of the field in which the herd was grazing before they were discovered and put out. This was the only way that field could have been infected. Of course, ticks could have been dropped on the pasture by these strange cattle, but, as I said before, none was found on Col. A.'s cattle. I, of course, attributed the infection to the excretions of the strange cattle.

After the outbreak of the disease the herd was removed from the pasture to the stable and stable lot, where it was kept until after cold weather had set in. In this outbreak the disease attacked young and old, cows that were born and raised on the place as well as animals from the North.

I fully believe that cattle born and raised on an infected farm acquire immunity from the disease, and I am quite sure that such immunized cattle convey the infection to non-infected pastures, and it is by this means only that the infected area is extended or perpetuated.

Cattle born and raised on a non-infected farm sicken and probably die when put on an infected farm, although the infected farm may be only on the other side of a fence or on the opposite side of a road.

So far as I know, there is but one way to tell an infected farm from a non-infected farm, and that is by testing the pastures in the proper season, by placing on them cattle from a farm known to be non-infected.

Richmond City and the adjoining counties have been within the so-called infected area since the line was mapped out, and that there are many non-infected farms and sections in the vicinity is proved by the fact that hundreds of cattle born and raised on these farms die annually when brought to Richmond and turned out to pasture on the commons during the day, where they contract the disease.

Animals placed in a dairy stable where the cattle are stall-fed entirely, and have only the run of a yard wherein there is no grazing, neither infect others nor are liable to infection, no matter where they are born and raised.

To illustrate better some of the points I have called your attention to I will read a letter from Mr. W. E. Grant:

GRANTLAND, VA., December 13, 1894.

DR. W. H. HARBAUGH, Richmond, Va.

DEAR SIR: Your letter of the 12th inst. to hand. I have never seen a case of Southern fever, except at Col. —, where you were then in attendance, As you know, my place is above, and on the opposite side of the James River. I have never heard of a case in my immediate neighborhood, though on the opposite side of the river I have heard great complaints of sickness of this nature, I suppose. Cattle which I have sold to go to Chesterfield have died, though in perfect health when I delivered them.

I have brought cattle to my place from the mountains of North Carolina and Virginia, New York, Pennsylvania, Holland, and almost direct from the Island of Jersey. I would not hesitate to bring cattle from any place on the face of the earth to my farm if healthy at the time of removal. I have often seen ticks on my cattle, but am not aware that they did any especial injury.

My farm is fenced, but other people's cattle often get in with mine (I don't like to own it, but mine sometimes return their visits).

My cattle are out in the pasture every day in the year, unless there is falling weather or it is extremely cold. I have been, and am, very careful that my cattle shall never have access to stagnant water. This is the only precaution I have ever deemed necessary.

James River is practically an insurmountable barrier between the two counties; therefore, I know very little about Chesterfield County, but I have always understood that cattle in that county had but few restrictions placed upon them. It is unfortunate that so large a portion of the United States should be placed under a ban simply because a few places within that limit have been found unhealthful. Yours, truly,

(Signed) W. E. GRANT.

Mr. Grant is a well-known gentleman, a reliable and practical breeder of Holstein and Jersey cattle, and implicit confidence may be placed in his statement. His farm, Grantland, is about five miles west of Richmond, in Henrico County, and in the so-called permanently infected district.

You must bear in mind that within the so-called infected area a cow born and raised on an infected farm, although insusceptible to the disease, and apparently healthy in every respect, will infect a non-infected pasture by grazing in it; and that a cow born and raised on a non-infected farm will contract the disease while grazing on an infected farm, and that an infected field may adjoin a non-infected field.

These are curious facts, but facts that are as well known to the practitioner as the unmistakable symptoms of the disease. Of course, there may be isolated instances which might be quoted as exceptions.

It is not my intention to occupy much of your time with the tick theory. I do not question any of the discoveries made by the bureau investigators, as far as they go; but you must remember that those experiments were conducted outside the so-called infected area, and seemingly with the object of connecting the tick with the cause of the disease.

Important as are their discoveries, there are many missing links, and while they have placed about all the responsibility on the tick at the experiment station, I am still of opinion that there are other factors as important, if not more so, in the so-called infected area, where the disease occurs seemingly without ticks, and where it does not occur in spite of ticks.

However, the tick theory should not be discarded. It should be carefully considered and intelligently understood, and if your observations in practice support it, you should not hesitate to confirm it as one of the factors that spread the infection in this

State. More good can be done by assisting the bureau in its efforts to solve doubtful points than by placing obstacles in its way, or by trying to prove it wrong.

I have perfect faith in the bureau's discovery of the protozoal micro-organisms in the red blood-corpuscles, because it thoroughly explains every phenomenon of the disease as well as the post-mortem lesions. But from whence comes the micro-organism? From the South, certainly. But how does it gain access to the blood of cattle? Does the tick get it from the infected cattle, or do the cattle get it originally from the tick? That the tick is not a necessary bearer of the microparasite is proved by the bureau's experiments of transmitting the disease by the transference of blood direct from a southern to a northern cow. We are told that the disease is spread by the young ticks. The old tick drops off the southern cow, lays its eggs, the eggs are hatched, and the young ticks crawl on susceptible cattle and cause the disease. Where do these young ticks get the protozoa? Again we are told "the young ticks, as they are hatched near the dead body of the female, may become infected from this." But this idea is negatived by the fact that they produce the disease with the young ticks artificially hatched. Another strange result of its experiments is noted as follows: "The crushed ticks introduced into the blood fail to produce any infection whatever, while ticks from the same lots when placed on the skin produce Texas fever."

The biological part of this subject must be left to the skilled specialist in that field, who, with his perfectly equipped laboratory, may in time settle some if not all of the doubtful points. But I suggest that the proper course for us to pursue is to consider ourselves a committee of observation, and that it be the duty of each member carefully to note every particular in each case treated, including a history of the animal as detailed as it is possible to obtain, and report the same in proper form, so that the recorded observations may be compiled into a paper to be read before the Association next fall or winter.

During the coming season I hope to be able to settle some of the doubtful points we meet with in practice, and, with the assistance of Prof. Niles and Prof. Hoge, I see no reason why we should not conduct some profitable experiments. Surely no Association is better situated to study the disease.

But little attention has been paid to the medical treatment of the disease, which to us, as practitioners, is of the greatest immediate importance. The investigators have devoted their time to the cause and nature of it.

In the winter of 1886 and 1887 I was in the vicinity of Albemarle Sound, in North Carolina, getting information about a horse disease for the bureau. Naturally, I conversed with a number of physicians of that section, and was informed by them of a serious and frequently fatal type of malaria in the human being they had to contend with, called by them hemorrhagic malarial fever. I was struck with the similarity of the symptoms to those of southern cattle fever. In fact, I know of no two diseases of man and the lower animals so much alike as these two. I wrote the particulars to a veterinarian, who was an authority on southern cattle fever, but he attached no importance to the similarity of the symptoms, considering them merely coincidental. However, I determined to go deeper into the subject, and, consequently, I devoted much of my spare time to reading up the diseases. The more I read the more I became convinced that one who learns much by reading has much to unlearn when he arrives at the naked truth. Of one thing I became convinced, however, and that was that southern cattle fever was either a malarial affection or closely related to that class of disease.

During the discussion of this subject I will call your attention to the symptoms, post-mortem lesions, history, etc., of hemorrhagic malarial fever as compared with southern cattle fever, so that you will comprehend the grounds on which I based my opinion. You must remember that the protozoa, the microparasite, had not been discovered in the red blood-corpuscle of cattle affected with Texas fever at the time of which I speak, and when the discovery was announced, some years later, it only confirmed me in my opinion as to the nature of the disease.

I must mention that many of the physicians who had experience with hemorrhagic malarial fever considered it a new disease; one of them told me he treated the first case that ever appeared in that section, in 1866. But another physician claimed that it was not a new disease, and to prove it he referred to some records in a Quaker meeting-house in Perquimans County, N. C, as evidence that there were cases of it in that county at least fifty years before 1866. He said that, while it was called yellow fever in those records, there could be no doubt that it was hemorrhagic malarial fever, as the history of the case gave ample evidence. It should be remarked that down there

"yellow chill" is a common name for the affection even at this date.

While there I learned that the majority of the physicians with whom I conversed on the subject depended on calomel and quinine as their remedial agents, and I determined to try the same treatment, as far as applicable, to southern cattle fever in the future.

During the following seasons my success was not what I had expected, but still I was not entirely discouraged, as I knew the mortality in the human disease often exceeded twenty-five per cent. I concluded I was giving quinine in too small doses, consequently I laid in a supply of quinine at wholesale prices, and gave it in wholesale doses, and my success became more marked; and now, after years of trial, I am satisfied that quinine is the proper antidote when properly administered and when given to the animal before dissolution has set in.

Although we are called to treat many hopeless cases of the disease, it by no means follows that all acute cases are hopeless. In many instances we are not called to treat ordinary sporadic diseases till they are both helpless and hopeless. The progress of the disease is so rapid that the case is often hopeless when the animal is discovered to be ill; but, fortunately, all acute cases do not become hopeless so rapidly.

I will simply outline my course of treatment in this communication, as we will go fully into the details of it in the discussion which will follow.

I first secure a comfortable stall with desirable surroundings (I never leave an affected animal on pasture if it is to be treated). I then administer a maximum dose of calomel; and in some cases combine other purgatives with it. I then prescribe extra large doses of sulphate of quinine every three or four hours till there is a marked fall in the temperature, or (if the animal is where I cannot see it frequently to take the temperature) until the urine becomes markedly more natural in color. I then continue the quinine, but increase the intervals between the doses as the temperature falls and the urine becomes natural in color.

I allow the animal all the cold drinking-water desired, and feed bran and meal slops if the animal will eat them, but no harm follows the practice of allowing any cured food the patient will eat. Some will nibble at hay, others blade fodder, and some will refuse all food. Such patients I drench with raw eggs and fresh milk, and sometimes with oatmeal gruel.

When the temperature has been reduced to 103° F., or lower, or when the urine is about natural in color, I stop the quinine and give a compound of arsenious acid, sulphate of iron, and nux vomica twice a day, and continue it so long as the cow remains debilitated or in bad condition.

In some cases you will have relapses, when you will have to resort to quinine again. Some cases recover rapidly and permanently, while some few cases only partially recover, linger for a month or two and die, apparently from malnutrition, even weeks after severe frosts. This latter termination is especially marked in cases where owners will persist in turning on pasture after the urgent symptoms have disappeared. Therefore, I advise that all recovered cases be kept off pasture till after cold weather has set in.

In recent years I look for ticks as a matter of curiosity. Sometimes I find them on patients, and sometimes I fail to find them, but I make no systematic search for them.

Such is an outline of my course of treatment, which, of course, is often modified when deemed necessary.

In the case of a human being with a serious disease there are premonitory symptoms which necessitate the calling of a physician before the alarming symptoms have appeared, but in the case of a cow the alarming symptoms have appeared and the disease has made a terrible onslaught on the vital organs before she is discovered to be ill; then we are called. With these facts in mind I have been comparatively satisfied with my average success.

When you have the disease to break out in a herd, one or more acute cases causes the alarm. If you will have all the cattle placed in the stable, so that you can take their temperature, you will often discover quite a percentage of them to be affected, showing no other symptons than an abnormal rise in temperature. Hence the necessity of this procedure.

I will cite two instances that occurred in my practice during the past season as examples covering many of the points referred to in the foregoing pages.

On July 16th, last, I was called to see a case below the city. This cow was one that was born and raised on Col. A.'s place in Chesterfield, before referred to. Her temperature was 107° F., and her urine dark in color. I prescribed the usual remedies, and she made a good recovery in about two weeks. On August 10th I was called to see her again, and found her suffering from an ordinary attack of laryngitis, from which she recovered in a few days. On August 28th I passed the farm and saw her grazing along the road; she was in good condition and was giving a fair quantity of milk. On October 8th and 15th I was called to prescribe for her for an obstinate diarrhœa. At this time there were no symptoms of southern cattle fever; her symptoms were characteristic of tuberculosis She died some weeks later, the exact date I was not informed, and consequently I had no opportunity of holding a post-mortem examination.

When I was first called to this cow I looked over her, but found no ticks. I asked the stableman if he had seen any on her. He said that he had removed a few very large ones a few days before.

On September 19th I was called to the farm of Mr. F., in Chesterfield County. I found three fine Jersey cows affected with southern cattle fever. Two were down, their urine was almost black, the temperature of both was 108° F. The other was standing, urine slightly tinged with coloring-matter of the blood, temperature 104° F. I discovered no ticks on them. The mild case and one of the acute cases recovered, the other died within twenty-four hours. It was the youngest cow that had the mild case.

All of these cows were born and raised on the farm of Mr. Grant, whose letter I have read to you. They were purchased by Mr. F. and taken to his place early in the spring, and did well until attacked by this fever in the latter half of September.

There is one very important point on which I have not been able to arrive at any conclusion whatever, and that is, whether or not an infected farm may in course of time become a non-infected farm.

We know that Mr. Grant's farm has ever been a non-infected farm, although his cattle have ticks on them; and I know of farms where the disease occurs year after year when new cattle are placed on them, and the question that occurs to my mind is: If all cattle are kept off such a place for a certain number of years, would that place become a safe farm for cattle raised on non-infected farms or cattle from without the so-called infected area?

At present I see no hope of success for preventive inoculation, although several experimenters think they have reached that point.

Natural immunity is acquired by cattle born and raised on an infected farm. If I intended to embark in the raising of valuable cattle, I would begin with cattle raised on an infected farm, and would buy an infected farm, and I would introduce more cattle from infected farms to keep my supply of infection, as it were, and I would be certain that my place was an infected place by placing on its pastures yearly one or more cheap cows from non-infected places as a test. Hence my breed of cattle would be insusceptible and healthy so far as this disease is concerned, and I would run no risk of such heavy losses as incurred by Col. A. These cattle would be safe to go to any infected farm in the South, and cattle of known immunity are needed, but, of course, they would carry destruction to non-infected places north or south of the line in the fever season.

